# The entertainment value of conspiracy theories

**DOI:** 10.1111/bjop.12522

**Published:** 2021-07-14

**Authors:** Jan‐Willem van Prooijen, Joline Ligthart, Sabine Rosema, Yang Xu

**Affiliations:** ^1^ VU Amsterdam The Netherlands; ^2^ The Netherlands Institute for the Study of Crime and Law Enforcement (NSCR) Amsterdam The Netherlands

**Keywords:** conspiracy theories, emotions, emotional intensity, entertainment, sensation seeking

## Abstract

Many citizens around the globe believe conspiracy theories. Why are conspiracy theories so appealing? Here, we propose that conspiracy theories elicit *intense* emotions independent of emotional valence. People therefore find conspiracy theories entertaining – that is, narratives that people perceive as interesting, exciting, and attention‐grabbing – and such entertainment appraisals are positively associated with belief in them. Five studies supported these ideas. Participants were exposed to either a conspiratorial or a non‐conspiratorial text about the Notre Dame fire (Study 1) or the death of Jeffrey Epstein (preregistered Study 2). The conspiratorial text elicited stronger entertainment appraisals and intense emotions (independent of emotional valence) than the non‐conspiratorial text; moreover, entertainment appraisals mediated the effects of the manipulation on conspiracy beliefs. Study 3 indicated that participants endorsed stronger conspiracy beliefs when an election event was described in an entertaining rather than a boring manner. Subsequent findings revealed that both organisational (Study 4) and societal conspiracy beliefs (Study 5) are positively associated with sensation seeking – a trait characterised by a preference for exciting and intense experiences. We conclude that one reason why people believe conspiracy theories is because they find them entertaining.

## Background

The Internet and social media are full of conspiracy theories such as that the CIA was behind the JFK assassination, that the virus causing Covid‐19 was developed by humans in a laboratory, and that NASA fabricates evidence that the Earth is round instead of flat (e.g., Butter & Knight, 2020; Grebe & Nattrass, 2012; Oliver & Wood, 2014; Sunstein & Vermeule, 2009; Van Prooijen, 2018). A conspiracy theory is defined as an explanatory belief involving multiple actors that collude in secret agreement to pursue malevolent goals (Bale, 2007). Conspiracy theories are not harmless: Large numbers of regular, well‐functioning citizens believe them, yet they are largely detrimental for people’s well‐being and psychological functioning. For instance, conspiracy theories are associated with anxiety (Grzesiak‐Feldman, 2013), self‐uncertainty (Van Prooijen, 2016), anomie (Goertzel, 1994), and feelings of powerlessness (Abalakina‐Paap, Stephan, Craig, & Gregory, 1999). Moreover, conspiracy beliefs can hurt people’s health (e.g., vaccine refusals; decreased social distancing during the Covid‐19 pandemic) and society (e.g., climate change denialism; intergroup conflict; crime; reduced prosocial behaviour; e.g., Imhoff & Lamberty, 2020; Jolley & Douglas, 2014a, 2014b; Jolley, Douglas, Leite, & Schrader, 2019; Van der Linden, 2015; Van Prooijen & Douglas, 2018; Van Prooijen & Van Vugt, 2018). These observations suggest a paradox: If conspiracy theories are detrimental for perceivers and their social environment, then why do so many people believe them? This research will illuminate that conspiracy beliefs have previously unrecognised psychological benefits to perceivers: Conspiracy theories are appealing because they have entertainment value.

Common explanations of belief in conspiracy theories have emphasised a central role of feelings of anxiety, uncontrollability, and uncertainty. Throughout history, societal crisis situations have increased conspiracy beliefs (Van Prooijen & Douglas, 2017), and various theoretical frameworks have articulated that the aversive feelings associated with distressing societal events increases an epistemic sense‐making process that facilitates conspiracy thinking (Douglas, Sutton, & Cichocka, 2017; Van Prooijen, 2020). Furthermore, evolutionary perspectives have proposed that such negative feelings activate an adaptive mental mechanism to protect against social threats, leading people to overestimate the likelihood that others are forming hostile coalitions (Raihani & Bell, 2018; Van Prooijen & Van Vugt, 2018). Correspondingly, empirical research has established that feelings of anxiety, uncontrollability, or affective uncertainty predict increased conspiracy beliefs (e.g., Grzesiak‐Feldman, 2013; Kofta, Soral, & Bilewicz, 2020; Newheiser, Farias, & Tausch, 2011; Van Prooijen, 2016; Van Prooijen & Acker, 2015; Whitson & Galinsky, 2008). Moreover, conspiracy theories are not functional to reduce such negative feelings; instead, conspiracy beliefs only increase such feelings (Douglas et al., 2017; Van Prooijen, 2020).

The line of reasoning underlying these theoretical insights and empirical findings includes a tacit assumption that is common within the psychology of conspiracy theories: The assumptions that feelings of anxiety, uncontrollability, and uncertainty necessarily always are aversive, and need to be managed psychologically (cf. Park, 2010; Van den Bos, 2009). This assumption provides a limited perspective, however, as often people display a preference for stimuli that elicit these feelings. For instance, a classic insight in psychology is that human performance can be impaired by stress levels that are not only too high, but also too low (Yerkes & Dodson, 1908). Moreover, people are risk‐averse in some situations yet risk‐seeking in others, implying a willingness to accept some levels of uncontrollability and uncertainty (Kahneman & Tversky, 1979). Finally, personality psychologists have recognised that people vary in sensation‐seeking, ‘a trait defined by the seeking of varied, novel, complex, and intense sensations and experiences, and the willingness to take physical, social, legal, and financial risks for the sake of such experiences’ (Zuckerman, 1994, p. 27). This trait implies that some people find anxiety‐provoking and uncertain situations rewarding, which pertains not only to genuinely risky behaviours (e.g., extreme sports; gambling) but also to risk‐free activities that bring excitement, novelty, and intense emotions such as attending scary movies, playing chess, and talking about science (Hwang & Southwell, 2007; Joireman, Fick, & Anderson, 2002; Morris & Griffiths, 2013; Roberti, 2004; Zuckerman, 1994).

Here, we propose that in a similar vein, conspiracy theories can be appealing to perceivers. Much like a scary movie or detective novel, conspiracy theories typically involve spectacular narratives that include mystery, suspected danger, and unknown forces that one does not fully comprehend (Van Prooijen, 2018). Jointly, these features can make learning about a conspiracy theory a fascinating and emotion‐arousing experience. Put differently, many conspiracy theories have potential *entertainment value*, which we define as the extent to which people appraise a particular narrative as interesting, exciting, and attention‐grabbing. Of importance, such entertainment appraisals can sometimes be related with emotions traditionally seen as negative: Scary movies often install anxiety in people, yet people find them entertaining, and pay money to see them. Instead, the entertaining qualities of conspiracy theories are likely associated with *intense* emotional experiences that can be negative, positive, or both, in valence.

### Emotional intensity and conspiracy beliefs

Previous research has predominantly conceptualised emotional intensity as a stable individual difference variable, defined as the intensity by which people tend to experience both negative and positive emotions independent of their frequency (e.g., Fujita, Diener, & Sandvik, 1991; Larsen & Diener, 1987). Situations also differ in their potential to elicit intense emotions, however, which has profound psychological implications. For instance, people experience less psychological distance towards events that elicit more intense emotions (Van Boven, Kane, McGraw, & Dale, 2010). Previous findings also suggest a role of emotional intensity for conspiracy beliefs. For instance, not only negative but also positive emotions increase conspiracy beliefs (Whitson, Galinsky, & Kay, 2015), and making events more emotionally involving through a perspective‐taking manipulation increases conspiracy beliefs as compared to a less emotionally involving control condition (Van Prooijen & Van Dijk, 2014). Furthermore, stability of self‐esteem (i.e., the extent to which it fluctuates over time) is a better predictor of conspiracy beliefs than level of self‐esteem (Van Prooijen, 2016).

These considerations suggest that conspiracy theories are associated with intense emotional experiences that are not exclusively negative. For instance, a perceiver may believe to be the first to discover an important secret with far‐ranging implications, and therefore experience a sense of meaning and purpose. Consistent with this line of reasoning, conspiracy beliefs are correlated with susceptibility to boredom (Brotherton & Eser, 2015), a need to feel unique and special (Imhoff & Lamberty, 2017; Lantian, Muller, Nurra, & Douglas, 2017) and narcissism (Cichocka, Marchlewska, & Golec de Zavala, 2016). At the group level, there is a robust link between conspiracy beliefs and collective narcissism, defined as an exaggerated esteem of the ingroup (Golec de Zavala & Federico, 2018). Finally, ambivalence – which includes both negative and positive feelings towards attitude objects – increases conspiracy beliefs (Van Harreveld, Rutjens, Schneider, Nohlen, & Keskinis, 2014). These insights are consistent with the notion that conspiracy theories are associated with intense emotional experiences.

The entertainment value of conspiracy theories suggests a novel explanation for the well‐known finding that exposure to conspiracy theories increases belief in them (Jolley & Douglas, 2014b). We assert that entertainment appraisals are associated with increased conspiracy belief. Theorising on the fluency heuristic stipulates that it is easier for people to mentally process information that is captivating and attention‐grabbing, which increases the likelihood of accepting such information as true (e.g., Brashier & Marsh, 2020). For instance, facilitating ease of processing by repeatedly exposing people to statements (Dechêne, Stahl, Hansen, & Wänke, 2010), or making statements more attention‐grabbing by presenting them in high contrast (e.g., bold fonts; Reber & Schwarz, 1999) have been found to increase truth judgments. Relatedly, intense emotional experiences are likely to activate System‐1 (i.e., automatic, intuitive, emotional) thinking and suppress System‐2 (i.e., deliberative, analytic) thinking (Kahneman, 2011). This is important, as conspiracy beliefs are associated with decreased analytic thinking, and an increased reliance on one’s emotions and intuitions (Swami, Voracek, Stieger, Tran, & Furnham, 2014). Taken together, these arguments are consistent with the notion that entertainment appraisals predict increased belief in conspiracy theories.

### Research overview

The current contribution includes five studies that have examined the entertaining qualities of conspiracy theories. Study 1 manipulated whether participants were exposed to a narrative supporting a conspiracy theory about a salient event in recent history (i.e., the fire of the Notre Dame cathedral in Paris, 2019), or a (syntactically comparable) narrative supporting the official, non‐conspiratorial explanation. The main prediction was that participants would find the conspiratorial narrative more entertaining than the non‐conspiratorial narrative. Besides entertainment appraisals, the study also assessed participants’ emotional experiences. Participants indicated not only if they experienced positive or negative emotions when reading the narrative (i.e., emotional valence), but also, how intense their emotions were independent of valence (i.e., emotional intensity). Based on the line of reasoning presented earlier, we expected a conspiracy theory to elicit more intense emotional experiences independent of emotional valence. Finally, we predicted that entertainment appraisals and emotional intensity would mediate the effect of conspiracy exposure on conspiracy belief.

Study 2 is a preregistered conceptual replication of Study 1, while manipulating exposure to a different conspiracy theory (i.e., the conspiracy theory that the convicted sex offender Jeffrey Epstein was murdered in his jail cell, versus the official reading that he committed suicide). Study 3, then, manipulated the mediator of Studies 1 and 2: participants were exposed to a narrative describing an election event, and we manipulated how entertaining or boring the narrative was by using emotionally intense or detached language. Studies 4 and 5 focused on the personality trait sensation seeking, commonly associated with a preference for exciting and intense experiences (Zuckerman, 1994). We tested if individual differences in sensation seeking would predict increased conspiracy beliefs; in Study 4 within the specific setting of organisations (Van Prooijen & De Vries, 2016; see also Douglas & Leite, 2017), and in Study 5 within the context of a range of common societal conspiracy theories (e.g., about the 9/11 terrorist strikes; the moon landings; Van Prooijen, Douglas, & De Inocencio, 2018).

### Open practices statement

All data and materials of the studies reported here, and the preregistration of Study 2, are publicly available on the Open Science Framework (https://osf.io/w7ekr/). For all the studies we report all the conditions and measures (either in the method sections or the Supplementary Materials); there were no data exclusions. The studies reported here have formal ethical approval (as part of an institutional cluster application by the first author) and were conducted in accordance with the provisions of the declaration of Helsinki.

## Study 1

### Method

#### Participants and design

We recruited 300 UK participants through Prolific (86 men, 214 women; *M*_age_ = 35.40, *SD* = 12.73), who were randomly assigned to one of the two conspiracy exposure conditions (conspiracy vs. control). This sample yields 90% power to detect a small‐to‐medium effect size (*d* = .37, two‐sided; approximately the equivalent of ω^2^ = .03). The study lasted about 10 min.

#### Procedure

Participants read an Internet article by an unknown writer (in fact experimenter‐designed) about the Notre Dame fire in Paris on 15 April 2019. In the *conspiracy* condition, participants read a narrative supporting the theory that the Notre Dame was set on fire deliberately, and that the truth was hidden from the public. In the *control* condition, participants read a narrative supporting the official reading that the Notre Dame fire was a tragic accident, and that all the relevant information to understand what happened that day has been shared with the public. The two conditions had an exact equal number of words, and followed the same syntactic and narrative structure; full texts in the Supplementary Materials. Participants were asked to briefly summarize the article, and as a check were asked dichotomously whether the writer believed that the Notre Dame fire was an accident.

Participants then completed a measure of how entertaining they rated the article on the following 12 dimensions (1 = *Not at all*, 5 = *Very much*): Interesting, entertaining, important, engaging, boring (recoded), mysterious, adventurous, dull (recoded), captivating, exciting, attention‐grabbing, and frightening. Participants’ responses were averaged into a reliable scale of entertainment appraisals (α = .91).^1^

We then asked participants to indicate on a slider how positive or negative the emotions were that they felt while reading the article (0 = *Very negative*, 100 = *Very positive*), and how intense their emotions were (regardless of whether they were positive or negative; 0 = *Not at all intense*, 100 = *Very intense*). Finally, we measured participants’ belief in a Notre Dame conspiracy theory with three items (1 = *Not at all,* 5 = *Very much*), for example, ‘Do you believe that a conspiracy was behind the Notre Dame fire?’ (α = .93). At the end of the study, participants provided basic demographics, were debriefed, and were redirected to a completion URL for payment.

### Results

The means, standard deviations, and intercorrelations are displayed in Table 1. Entertainment appraisals are substantially correlated with the intensity and not the valence of emotions. Moreover, entertainment appraisals and emotional intensity were both more strongly correlated with conspiracy beliefs than emotional valence.

**Table 1:** Means, standard deviations, and intercorrelations of the measured variables – Study 1

	Overall sample		Conspiracy condition		Control condition		Correlation table			
	*M*	*SD*	*M*	*SD*	*M*	*SD*	1	2	3	4
1. Entertainment appraisals	3.08	0.81	3.42	0.71	2.72	0.76	–			
2. Emotional valence	46.20	19.99	38.74	17.82	54.23	19.11	.04	–		
3. Emotional intensity	42.12	24.40	46.01	24.38	37.97	23.81	.52***	.02	–	
4. Belief in conspiracy theories	2.30	1.16	2.84	1.13	1.72	0.89	.44***	−.15**	.34***	–

Note

Entertainment appraisals and belief in conspiracy theories were measured on five‐point scales, emotional valence and emotional intensity on 100‐point scales. Higher means represent higher scores on the variable in question.

** *p* < .01; ***p < .001.

#### Manipulation check

Results revealed that 96.2% of the participants in the conspiracy condition and 93.1% in the control condition correctly identified whether or not the writer believed that the Notre Dame fire was an accident. These results indicate that participants perceived the manipulation correctly. Below are the results for the full sample; results after excluding participants who failed the manipulation checks are similar, and reported in the Supplementary Materials.

#### Dependent variables

We analysed the dependent variables with a series of ANOVAs. Degrees of freedom differ slightly across measures (and from the final sample) due to attrition during the study. Supporting our predictions, participants appraised the conspiracy text as more entertaining than the control text, *F*(1, 300) = 68.54, *p* < .001; ω^2^ = .18, CI_95%_[0.11; 0.27] (means and standard deviations in Table 1). Furthermore, participants experienced more negative emotions when reading the conspiracy as opposed to the control text, *F*(1, 299) = 53.02, *p* < .001; ω^2^ = .15, CI_95%_[0.08; 0.23]. We then analysed emotional intensity while statistically controlling for emotional valence. Results revealed that emotional valence was not a significant covariate, *F*(1, 297) = 2.74, *p* = .099; ω^2^ = .01, CI_95%_[0.00; 0.04], and that participants reported more intense emotions in the conspiracy than the control condition, *F*(1, 297) = 10.97, *p* = .001; ω^2^ = .03, CI_95%_[0.00; 0.08]. Finally, consistent with previous research (Jolley & Douglas, 2014b; Jolley et al., 2019), exposing participants to a conspiracy theory increased their belief in it, *F*(1, 298) = 91.42, *p* < .001; ω^2^ = .23, CI_95%_[0.15; 0.32].

#### Mediational analysis

**Figure 1:**
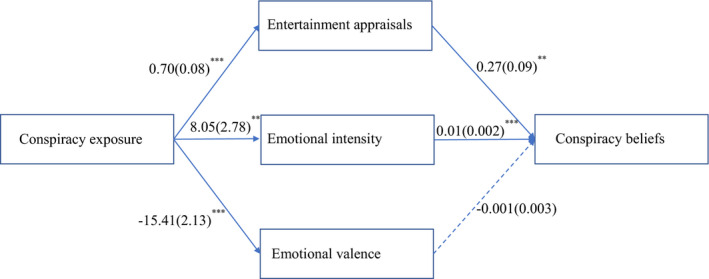
Mediation model Study 1. Coefficients are *B*(*SE*). **p < .01; ***p < .001. Dotted line is non‐significant (*p* = .69).

We then tested whether the effects of conspiracy exposure on conspiracy beliefs were mediated by entertainment appraisals, emotional valence, and emotional intensity. In a bootstrapping analysis (PROCESS model 4; 1,000 samples, bias‐corrected; Hayes, 2013), we entered these variables simultaneously as three parallel mediators. The results are displayed in Figure 1. The indirect effect through entertainment appraisals was significant, *B* = 0.19, *SE* = .07; CI_95%_[0.05; 0.34], as was the indirect effect through emotional intensity, *B* = 0.07, *SE* = .03; CI_95%_[0.02; 0.16]. The indirect effect through emotional valence was non‐significant, *B* = 0.02, *SE* = .06; CI_95%_[−0.09; 0.15]. These findings suggest that exposure to a conspiracy theory increases belief in it due to entertainment appraisals and emotional intensity, and not due to negative emotional valence.

### Discussion

Study 1 provided preliminary support for the entertainment value of conspiracy theories. Participants rated a Notre Dame conspiracy theory as more entertaining, and more emotionally intense, than a narrative supporting the official reading of this event. Moreover, entertainment appraisals and emotional intensity mediated the effects of conspiracy exposure on conspiracy beliefs.

## Study 2

Study 2 was designed to replicate and extend the Study 1 findings. To exclude the possibility that the Study 1 findings are due to idiosyncrasies of the Notre Dame fire, in Study 2 we focused on a different conspiracy theory, namely that the wealthy US financier and convicted sex offender Jeffrey Epstein was murdered in his jail cell by powerful people. Moreover, while Study 1 took place among UK participants, we conducted Study 2 among US participants. Finally, we preregistered the design, hypothesis, and analysis plan of Study 2 on the Open Science Framework before running it. Our preregistered hypotheses were that participants would rate the conspiracy text as more entertaining than the control text (Hypothesis 1); that the conspiracy text would elicit more intense emotions than the control text, independent of (and after controlling for) emotional valence (Hypothesis 2); and that entertainment and emotional intensity would mediate the effects of conspiracy exposure on conspiracy beliefs, independent of emotional valence (Hypothesis 3).

### Method

#### Participants and design

We conducted the study among 301 US participants through Prolific (146 men, 155 women; *M*_age_ = 37.55, *SD* = 13.97), who were randomly assigned to one of the two conspiracy exposure conditions (conspiracy vs. control). This sample yields 90% power to detect a small‐to‐medium effect size (*d* = .38, two‐sided; approximately the equivalent of ω^2^ = .03). The study lasted about 10 min.

#### Procedure

Participants again read an Internet article by an unknown writer (in fact experimenter‐designed); however, in Study 2 this article pertained to the convicted sex offender Jeffrey Epstein, who died in his jail cell on 10 August 2019. In the *conspiracy* condition, participants read a narrative supporting the theory that Jeffrey Epstein was murdered by powerful people who feared a possible testimony. In the *control* condition, participants read a narrative supporting the official explanation that Jeffrey Epstein committed suicide. As in Study 1, the two conditions had an exact equal number of words, and followed the same syntactic and narrative structure (full texts in the Supplementary Materials). Participants were asked to briefly summarise the article, and as a check were asked dichotomously whether the writer believed that Jeffrey Epstein committed suicide.

Participants then responded to the items that comprised the dependent variables. The measures of entertainment appraisals (α = .92), emotional valence, and emotional intensity were the same as in Study 1. We assessed participants’ conspiracy beliefs with three items (1 = *Not at all*, 5 = *Very much*), for example, ‘Do you believe that Jeffrey Epstein was murdered by powerful people?’ (α = .91). Participants then provided basic demographics, were debriefed, and were redirected to a completion URL for payment.

### Results

The means, standard deviations, and intercorrelations are displayed in Table 2. As in Study 1, entertainment appraisals were strongly correlated with the intensity of emotions, although in this study the correlation with emotional valence was also significant. Moreover, entertainment appraisals and emotional intensity were significantly correlated with conspiracy beliefs, while emotional valence was not. Finally, the means suggested that on average, belief in this particular conspiracy theory was relatively high among participants, as the overall mean was significantly higher than the scale midpoint of 3, *t*(300) = 10.64, *p* < .001; *d* = .61.

**Table 2:** Means, standard deviations, and intercorrelations of the measured variables – Study 2

	Overall sample		Conspiracy condition		Control condition		Correlation table			
	*M*	*SD*	*M*	*SD*	*M*	*SD*	1	2	3	4
1. Entertainment appraisals	3.14	0.88	3.50	0.76	2.72	0.81	–			
2. Emotional valence	39.73	22.78	42.56	23.16	36.38	21.94	.23***	–		
3. Emotional intensity	47.31	27.62	52.69	24.85	40.95	29.41	.57***	.11^†^	–	
4. Belief in conspiracy theories	3.73	1.20	3.98	1.01	3.44	1.33	.28***	−.07	.17**	–

Note

Entertainment appraisals and belief in conspiracy theories were measured on five‐point scales, emotional valence and emotional intensity on 100‐point scales. Higher means represent higher scores on the variable in question.

^†^ *p* < .10; **p < .01; ***p < .001.

#### Manipulation check

A total of 88.3% of the participants in the conspiracy condition and 88.5% in the control condition correctly identified whether or not the writer believed that Jeffrey Epstein committed suicide. These results indicate that the manipulation was successful. Following our preregistered protocol, we report the results for the full sample; results after excluding participants who failed the manipulation check are similar, and reported in the Supplementary Materials.

#### Dependent variables

Confirming Hypothesis 1, participants rated the conspiracy text as more entertaining than the control text, *F*(1, 299) = 73.17, *p* < .001; ω^2^ = .19, CI_95%_[0.12; 0.28] (means and standard deviations in Table 2). Somewhat surprisingly (although not incompatible with our line of reasoning and preregistered hypotheses), participants experienced more *positive* emotions when reading the conspiracy as opposed to the control text, *F*(1, 299) = 5.60, *p* = .019; ω^2^ = .02, CI_95%_[0.00; 0.05]. Apparently, participants felt better reading about a sex offender being murdered than about a sex offender committing suicide.

We then analysed emotional intensity while statistically controlling for emotional valence. As in Study 1, emotional valence was not a significant covariate, *F*(1, 298) = 2.18, *p* = .141; ω^2^ = .00, CI_95%_[0.00; 0.03]. Supporting Hypothesis 2, participants reported more intense emotions in the conspiracy than the control condition, *F*(1, 298) = 12.43, *p* = .001; ω^2^ = .04, CI_95%_[0.01; 0.09]. Finally, exposing participants to a Jeffrey Epstein conspiracy theory increased their belief in it, *F*(1, 299) = 15.29, *p* < .001; ω^2^ = .05, CI_95%_[0.01; 0.10].

#### Mediational analysis

**Figure 2:**
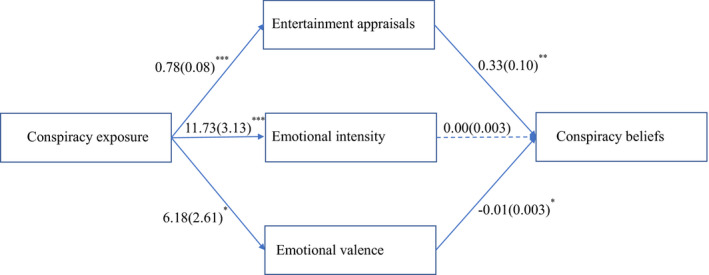
Mediation model Study 2. Coefficients are *B*(*SE*). *p < .05; **p < .01; *** *p* < .001. Dotted line is non‐significant (*p* = .78).

We then tested Hypothesis 3 that the effects of conspiracy exposure on conspiracy beliefs were mediated by entertainment appraisals and emotional intensity independent of emotional valence. Following our preregistered analysis plan, we entered these variables simultaneously as three parallel mediators in a bootstrapping analysis (PROCESS model 4; 1,000 samples, bias‐corrected; Hayes, 2013). The mediation model is displayed in Figure 2. The results partially supported Hypothesis 3: The indirect effect through entertainment appraisals was significant, *B* = 0.26, *SE* = .09; CI_95%_[0.10; 0.45], but the indirect effect through emotional intensity was not significant, *B* = 0.01, *SE* = .04; CI_95%_[−0.06; 0.09]. Unlike Study 1, the indirect effect through emotional valence was significant, *B* = −0.05, *SE* = .03; CI_95%_[−0.12; −0.01]. These findings further support our line of reasoning that exposure to a conspiracy theory increases conspiracy beliefs due to entertainment appraisals.

### Discussion

Study 2 largely replicated the Study 1 findings. Entertainment appraisals – although not emotional intensity – mediated the effects of conspiracy theory exposure on conspiracy beliefs.

## Study 3

A drawback of Studies 1 and 2 is that the link between entertainment appraisals and conspiracy beliefs is correlational. Study 3 was designed to provide evidence for a causal chain by manipulating the mediator of Studies 1 and 2 (Spencer, Zanna, & Fong, 2005): Participants read about an event that did not contain information about a conspiracy (i.e., an election event in a fictitious country), and we manipulated the entertainment value of the text by varying expressions of intense emotions. The prediction was that an entertaining text would elicit stronger conspiracy beliefs than a boring text. In addition, Study 3 explored the possible role of the personality trait sensation seeking. This personality trait directly assesses people’s preference for emotion‐arousing, exciting, and novel experiences (Zuckerman, 1994). We specifically assessed if (1) sensation seeking would be associated with conspiracy beliefs and (2) the links between conspiracy beliefs, intense emotional experiences, and entertainment appraisals would be particularly pronounced for participants high on sensation seeking.

### Method

#### Participants and design

We solicited 500 US participants through Prolific (258 men, 234 women, 8 other; *M*_age_ = 34.22, *SD* = 11.42). Participants were randomly assigned to one of the two entertainment value conditions (entertaining vs. boring). This sample provides 90% power to detect a small‐to‐medium effect size (*d* = .29, two‐sided; approximately the equivalent of ω^2^ = .02).

#### Procedure

Participants read a short text about an election event in a fictitious country (‘Contoria’). In the entertaining condition, the text described intense emotional experiences (e.g., ‘The two candidates disagree completely about many issues that are important for the future of Contoria, and they are extremely close to one another in the polls. In debates both candidates passionately argued for their ideas but can’t seem to agree on anything, and regularly they appear to be genuinely angry at each other’). In the boring condition, the same event was described using emotionally detached, bureaucratic language (e.g., ‘The two candidates have different positions about effective governance, and seem to have a comparable basis of electoral support. In debates both candidates exchanged their ideas of what legislation and law‐making institutions are in need of refinement, and made it apparent that they have different viewpoints on these issues’). Both conditions contained an exact same number of words, and are provided in the Supplementary Materials.

As manipulation checks, we measured entertainment appraisals (α = .92) and emotional intensity in the same manner as Studies 1 and 2, and again measured emotional valence as control variable. The dependent variable was belief in conspiracy theories. Participants rated how likely they considered seven items (1 = *very unlikely*, 5 = *very likely*), such as ‘There will be cheating in the results counting process’, and ‘A conspiracy will determine the election outcome’ (α = .96). Finally, we measured a short, validated 8‐item scale of sensation seeking (Hoyle, Stephenson, Palmgreen, Pugzles Lorch, & Donohew, 2002), for example, ‘I would like to explore strange places’, and ‘I get restless when I spend too much time at home’ (1 = *strongly disagree*, 5 = *strongly agree*; α = .83).^2^

### Results

#### Manipulation checks

Participants appraised the entertaining text as more entertaining (*M* = 3.05, *SD* = 0.78) than the boring text (*M* = 2.49, *SD* = 0.83), *F*(1, 498) = 60.286, *p* < .001; ω^2^ = .11, CI_95%_[0.06; 0.16]. Moreover, we analysed emotional intensity while controlling for emotional valence. Although emotional valence was a significant covariate, *F*(1, 497) = 20.278, *p* < .001; ω^2^ = .04, CI_95%_[0.01; 0.08], participants experienced more intense emotions after reading the entertaining text (*M* = 45.74, *SD* = 25.93) than after reading the boring text (*M* = 30.53, *SD* = 24.87), *F*(1, 497) = 61.037, *p* < .001; ω^2^ = .11, CI_95%_[0.06; 0.16]. These findings indicate that the manipulation of entertainment value was successful.

#### Conspiracy beliefs

The entertainment manipulation exerted a significant effect on conspiracy beliefs, *F*(1, 498) = 28.443, *p* < .001; ω^2^ = .05, CI_95%_[0.02; 0.10]. Participants believed conspiracy theories more strongly after reading an entertaining text (*M* = 2.48, *SD* = 1.10) than after reading a boring text (*M* = 2.01, *SD* = 1.00). This finding supports our prediction.

#### Sensation seeking

Results revealed a positive correlation between sensation seeking and conspiracy beliefs (*r* = .12, *p* = .010). We then explored if the relationship of conspiracy beliefs with entertainment appraisals and emotional intensity would be pronounced particularly among high sensation‐seekers. To this end, we entered the mean‐centered sensation‐seeking and conspiracy beliefs scales in Step 1 of hierarchical regression analyses, and their interaction in Step 2, with entertainment appraisals, and emotional intensity (controlling for emotional valence), as dependent variables. While Step 1 did not reveal a direct association of sensation seeking and entertainment appraisals (β = .013, *p* = .774) or emotional intensity (β = −.055, *p* = .220), consistent with Studies 1 and 2 the main effect of conspiracy beliefs was significant (for entertainment appraisals, β = .151, *p* < .001; for emotional intensity, β = .162, *p* < .001). More importantly, results revealed significant interactions (for entertainment appraisals, β = .099, *p* = .027; for emotional intensity, β = .101, *p* = .025). Among participants low in sensation seeking (−1*SD*), conspiracy beliefs were not associated with entertainment appraisals (β = .062, *p* = .305) or emotional intensity (β = .070, *p* = .244). Among participants high in sensation seeking (+1*SD*), however, conspiracy beliefs were significantly related with entertainment appraisals (β = .246, *p* < .001) and emotional intensity (β = .256, *p* < .001).

### Discussion

The results of Study 3 provided further evidence for the entertainment value of conspiracy theories: Participants formed stronger conspiracy beliefs in response to an entertaining than a boring text about an election event. Moreover, the results provided preliminary evidence that sensation seeking is positively correlated with conspiracy beliefs, and that the association of conspiracy beliefs with entertainment appraisals and emotional intensity emerges only among participants high in sensation seeking. In Studies 4 and 5, we more directly examine the role of sensation seeking in conspiracy beliefs.

## Study 4

Various studies underscore that sensation seeking is associated with closely related constructs: Specifically, supernatural beliefs elicit a sense of mystery, wonder, and excitement, and this makes them particularly appealing to people high in sensation seeking (Kumar, Pekala, & Cummings, 1993; Smith, Johnson, & Hathaway, 2009; Zuckerman, 1994). These insights are relevant as our line of reasoning suggests that conspiracy beliefs have similar entertaining qualities. Moreover, supernatural beliefs are positively correlated with conspiracy beliefs (Darwin, Neave, & Holmes, 2011; Lobato, Mendoza, Sims, & Chin, 2014; Van Prooijen et al., 2018).

Consistent with these arguments, one previous study found that conspiracy beliefs are related with susceptibility to boredom (Brotherton & Eser, 2015). Sensation seeking is a broader construct than boredom susceptibility, however, and no studies have yet examined its relationship with conspiracy beliefs. Specifically, sensation seeking has four underlying dimensions, namely boredom susceptibility (i.e., an aversion to repetition and routine), experience seeking (i.e., a desire to seek new experiences), disinhibition (a desire for social or sexual disinhibition), and thrill and adventure seeking (a desire for activities that involve speed or danger; Zuckerman, Eysenck, & Eysenck, 1978). We predicted that people believe conspiracy theories more strongly to the extent that they have stronger sensation‐seeking desires.

Study 4 examined organisational conspiracy theories among employees at the work floor. Organisational conspiracy theories are common, and are defined as beliefs among employees that their managers secretly conspire to pursue malevolent goals (Van Prooijen & De Vries, 2016; see also Douglas & Leite, 2017). Furthermore, we assessed participants’ conspiracy mentality as an additional dependent variable. Conspiracy mentality is a trait‐like predisposition to attribute events in the world to the causal actions of conspiracies (Bruder, Haffke, Neave, Nouripanah, & Imhoff, 2013; Imhoff & Bruder, 2014).

### Method

#### Participants and design

The study was conducted online, on a US sample through Amazon Mechanical Turk. Our sample contained 296 participants (139 women, 157 men, *M*_age_ = 34.01, *SD* = 9.26; 47.9%; Caucasian, 26.0%, Latin American, 7.2%, African American, 6.2% Native American, 3.4% Asian American, 9.2% other). This sample had 95% power to detect a small‐to‐medium effect size (*f* ^2^ = .04, which is the equivalent of *R* ^2^ = 0.038; α = .05). Both the study advertisement on Mturk and the informed consent page stated that, to participate in the survey, one had to work in an organisation of at least 15 employees, with at least one leader, and for at least 3 months. The study had a cross‐sectional design and consisted of a series of demographics including age, gender, education level (1 = *no formal education*, 5 = *college education [graduate degree]*), and political ideology (1 = *very left‐wing*, 11 = *very right‐wing*). After this, participants responded to a sensation seeking scale, a scale of organisational conspiracy beliefs, and a measure of conspiracy mentality.

#### Measures

Full scales are in the Supplementary Materials. *Sensation seeking* was measured with the SSS‐V developed by Zuckerman (1994).^3^ The scale consists of 40 items, each containing two statements (including one high and one low sensation‐seeking option), and participants’ task was to select what statement described their likes or feelings best. Items referred to the four dimensions of sensation seeking, notably boredom susceptibility (e.g., ‘There are some movies I enjoy seeing a second or even a third time’ vs. ‘I can’t stand watching a movie that I’ve seen before’), disinhibition (e.g., ‘I like “wild” uninhibited parties’ vs. ‘I prefer quiet parties with good conversation’), experience seeking (e.g., ‘I like to explore a strange city or section of town by myself, even if it means getting lost’ vs. ‘I prefer a guide when I am in a place I don’t know well’), and thrill and adventure seeking (e.g., ‘I often wish I could be a mountain climber’ vs. ‘I can’t understand people who risk their necks climbing mountains’). In keeping with previous research (e.g., Joireman et al., 2002; Kumar et al., 1993; Smith et al., 2009), we aggregated participants’ scores of all 40 items into one composite measure of sensation seeking (α = .75), while in our analyses also exploratively examining the four underlying dimensions separately.

To measure *organisational conspiracy beliefs,* we used the nine‐item scale by van Prooijen and de Vries (2016). Items included ‘Our management has a hidden agenda’ and ‘I suspect that our managers frequently lie to employees about important issues’ (1 = *Strongly disagree*, 5 = *Strongly agree;* α = .81).

Finally, we measured *conspiracy mentality* with the five‐item questionnaire by Bruder et al. (2013) with items such as ‘I think that many very important things happen to the world, which the public is never informed about’ (1 = *certainly not*, 11 = *certainly;* α = .93).

### Results

We analysed the results with hierarchical regression analyses. Given that Study 4 was cross‐sectional (unlike Studies 1 to 3 where participants were assigned randomly to conditions), Step 1 of the regression model included a range of control variables (age, gender, education level, and political ideology). Step 2 then added sensation seeking to the model. Degrees of freedom deviate from the total sample due to attrition and missing values. Results are displayed in Table 3.

**Table 3:** Results of hierarchical regression analyses: organisational conspiracy beliefs and conspiracy mentality as function of sensation seeking (Study 4)

	Organisational conspiracy beliefs			Conspiracy mentality		
	*B* (*SE*)	CI_95%_	β	*B* (*SE*)	CI_95%_	β
Step 1						
Gender	−0.16 (.09)	−0.34; 0.02	−.10	−0.38 (.29)	−0.95; 0.18	−.08
Age	−0.01 (.005)	−0.015; 0.004	−.07	0.02 (.02)	−0.01; 0.05	.07
Education	0.13 (.05)	0.02; 0.23	.14*	−0.15 (.17)	−0.48; 0.17	−.06
Political ideology	0.06 (.015)	0.03; 0.09	.24***	−0.06 (.05)	−0.15; 0.04	−.07
Step 2						
Sensation seeking	0.04 (.01)	0.03; 0.06	.32***	−0.03 (.03)	−0.08; 0.02	−.08

Note

*p < .05; **p < .01; ***p < .001.

#### Organisational conspiracy beliefs

Step 1 was significant, *F*(4, 277) = 8.73, *p* < .001; *R* ^2^ = .11, which was attributable to significant effects of political ideology and education level (See Table 3). Importantly, Step 2 was also significant *F*(1, 276) = 31.09, *p* < .001; Δ*R* ^2^ = .09. As predicted, sensation seeking positively predicted organisational conspiracy beliefs.

In an exploratory fashion, we also analysed the four subscales of this construct separately. Results revealed significant correlations of organisational conspiracy beliefs with boredom susceptibility (*r* = .36, *p* < .001), disinhibition (*r* = .22, *p* < .001), and thrill and adventure seeking (*r* = .22, *p* < .001) but not with experience seeking (*r* = .09, *p* = .12). These findings suggest that multiple dimensions of sensation seeking predict susceptibility to organisational conspiracy beliefs.

#### Conspiracy mentality

Both steps of the model were not significant, Step 1: *F*(4, 277) = 1.21, *p* = .31; *R* ^2^ = .02; Step 2: *F*(1, 276) = 1.61, *p* = .21; Δ*R* ^2^ = .01. Sensation seeking did not significantly predict conspiracy mentality. Of the four sensation‐seeking dimensions, conspiracy mentality was uncorrelated with disinhibition (*r* = −.07, *p* = .26), experience seeking (*r* = −.04, *p* = .54), and thrill and adventure seeking (*r* = .04, *p* = .49), and it was *negatively* correlated with boredom susceptibility (*r* = −.24, *p* < .001).

### Discussion

Study 4 supported the prediction that sensation‐seeking is associated with increased belief in organisational conspiracy theories. The study did not yield evidence for a relationship with conspiracy mentality, however. These findings suggest that the entertaining qualities of conspiracy theories only apply to concrete and specific conspiracy theories, not to a general predisposition to attribute events in the world to conspiracies.

## Study 5

The fifth and final study of this contribution was a conceptual replication of Study 4. Instead of organisational conspiracy theories, however, the study focused on the relationship of sensation seeking with belief in a range of well‐known societal conspiracy theories, such as about the 9/11 terrorist strikes and the Apollo moon landings (Van Prooijen et al., 2018).

### Method

#### Participants and design

This study was conducted among 410 American participants, again recruited through Amazon Mechanical Turk (169 women, 241 men; *M*_age_ = 38.00, *SD* = 11.12; 76.8% Caucasian, 4.6% Latin American, 10.5% African American, 1.7% Native American, 5.6% Asian American, 0.7% other). This sample yields 95% power to detect a relatively small effect size (*f* ^2^ = .03, which is the equivalent of *R^2^* = .029; α = .05). The study had a cross‐sectional design.

#### Measures

*Political ideology* was assessed with two items: ‘How would you describe yourself politically?’ (1 = *very left‐wing*, 11 = *very right‐wing*) and ‘Do you consider yourself to be a Democrat or a Republican?’ (1 = c*learly democrat, 11* = *clearly republican*). The two items were strongly correlated (*r* = .86, *p* < .001), and averaged into a reliable measure of political ideology.

*Sensation seeking* was measured with the same scale as in Study 4 (α = .84). To measure *conspiracy beliefs*, participants responded to the nine‐item scale by Van Prooijen et al. (2018). This scale assessed participants’ belief in nine specific conspiracy theories, including items such as ‘The US government had advance knowledge of the 9/11 attacks’ and ‘The moon landing was a hoax’ (1 = *Definitely not true*, 5 = *Definitely true*). Participants’ responses to these conspiracy theories together formed a reliable scale, and were averaged into a single index of conspiracy belief (α = .86). Finally, we measured conspiracy mentality with the same scale as Study 3 (Bruder et al., 2013; α = .90).

### Results

The data analytic strategy was the same as Study 4. Degrees of freedom again deviate from the total sample due to attrition and missing values. The regression results are displayed in Table 4.

**Table 4:** Results of hierarchical regression analyses: conspiracy beliefs and conspiracy mentality as function of sensation seeking (Study 5)

	Conspiracy beliefs			Conspiracy mentality		
	*B* (*SE*)	CI_95%_	β	*B* (*SE*)	CI_95%_	β
Step 1						
Gender	0.19 (.08)	0.02; 0.35	.11*	0.43 (.21)	0.006; 0.85	.10*
Age	−0.01 (.004)	−0.02; −0.004	−.15*	−0.02 (.009)	−0.04; 0.00	−.10*
Education	−0.07 (.05)	−0.17; 0.04	−.06	−0.18 (.14)	−0.45; 0.09	−.07
Political ideology	0.09 (.013)	0.06; 0.12	.32***	0.09 (.04)	0.02; 0.16	.12*
Step 2						
Sensation seeking	0.02 (.006)	0.01; 0.03	.18***	0.04 (.02)	0.005; 0.07	.12*

Note

*p < .05; **p < .01; ***p < .001.

#### Conspiracy beliefs

Step 1 of the regression model was significant, *F*(4, 395) = 14.03, *p* < .001; *R* ^2^ = .12. Age, gender, and political ideology all predicted conspiracy beliefs, such that younger age and right‐wing orientation predicted increased beliefs in conspiracy theories, and women reported slightly stronger conspiracy beliefs than men (*M*_women_ = 2.74, *SD* = 0.80; *M*_men_ = 2.65, *SD* = 0.89). More importantly, Step 2 was also significant, *F*(1, 394) = 14.48, *p* < .001; Δ*R* ^2^ = .03. Again, belief in conspiracy theories was associated with increased sensation seeking. Consistent with Study 3, conspiracy beliefs were significantly correlated with boredom susceptibility (*r* = .11, *p* = .026), disinhibition (*r* = .11, *p* = .026), and thrill and adventure seeking (*r* = .15, *p* = .003) but not with experience seeking (*r* = −.02, *p* = .74).

#### Conspiracy mentality

While in Study 4 no effects emerged for conspiracy mentality, in Study 5 both steps of the model were significant: for Step 1, *F*(4, 394) = 3.51, *p* = .008, *R* ^2^ = .03; for Step 2, *F*(1, 393) = 5.08, *p* = .025, Δ*R* ^2^ = .01. Conspiracy mentality was associated with younger age and right‐wing political orientation. Furthermore, women had slightly higher conspiracy mentality than men (*M*_women_ = 7.63, *SD* = 1.96; *M*_men_ = 7.30, *SD* = 2.19). More relevant for the present purposes, sensation seeking predicted increased conspiracy mentality. On the subdimensions, however, conspiracy mentality was significantly correlated only with disinhibition (*r* = .11, *p* = .026). It was uncorrelated with boredom susceptibility (*r* = .02, *p* = .63), experience seeking (*r* = .02, *p* = .66) and thrill and adventure seeking (*r* = .07, *p* = .18).

### Discussion

Consistent with the previous studies, sensation seeking predicted increased belief in a range of societal conspiracy theories. Moreover, this relationship was attributable to the same three underlying dimensions of sensation seeking as in Study 4 (i.e., boredom susceptibility, disinhibition, and thrill and adventure seeking). Unlike Study 4, the relationship of sensation seeking with conspiracy mentality was also significant. Closer inspection revealed that this finding was attributable to only one of the sensation‐seeking dimensions, however (i.e., disinhibition). In conjunction with the lack of an effect in Study 4, we therefore regard the link of sensation seeking with the broader trait conspiracy mentality as inconclusive at this point. Instead, the results reveal that sensation seeking reliably predicts people’s belief in specific and concrete conspiracy theories.

## General discussion

The psychology of belief in conspiracy theories suggests a paradox: Conspiracy beliefs have harmful implications for perceivers and their social environment, yet many people hold such beliefs (Butter & Knight, 2020; Douglas et al., 2017; Jolley & Douglas, 2014a, 2014b; Van Prooijen, 2018; Van Prooijen & Douglas, 2018). The present research sought to illuminate that conspiracy theories have a psychological payoff for perceivers: People often perceive conspiracy theories as entertaining, which facilitates belief in them. Results of five studies are consistent with this notion. Studies 1 and 2 manipulated exposure to a conspiracy theory (about the Notre Dame fire and Jeffrey Epstein), and results revealed that entertainment appraisals mediated the effects of conspiracy exposure on conspiracy belief. Study 3 manipulated how entertaining or boring a description of an election event was, and this manipulation shaped conspiracy beliefs. Studies 4 and 5 investigated the implications of these insights for the personality trait sensation seeking, which was associated with both belief in organisational conspiracy theories (Study 4) and societal conspiracy theories (Study 5). Together, these studies support the idea that conspiracy theories have entertainment value, which helps explain belief in such theories.

Three more specific theoretical contributions for the emerging research domain of conspiracy beliefs follow from the present research. First, the present studies provide a novel answer to the question why conspiracy theories are so widespread in society. While we do not dispute that, quite often, conspiracy theories emerge from aversive experiences (e.g., societal crisis situations), the present studies expand on a range of recent finding suggesting that conspiracy beliefs sometimes also may have psychological benefits: Belief in conspiracy theories is associated with feeling unique and special (Imhoff & Lamberty, 2017; Lantian et al., 2017), an inflated evaluation of the self (i.e., narcissism; Cichocka et al., 2016) and an inflated evaluation of the groups that are central to a perceiver’s identity (i.e., collective narcissism; Golec de Zavala & Cichocka, 2012; Golec de Zavala & Federico, 2018). But while these previous findings clarify how conspiracy theories may be positively related with people’s self‐perception and identity, the present research adds to these findings by revealing that people also may perceive conspiracy theories as entertaining – that is, interesting, exciting, and attention‐grabbing narratives.

Second, and relatedly, the present research suggests that not necessarily negative emotions, but rather, *intense* emotional experiences predict conspiracy beliefs. While the majority of studies have focused on negative feelings and emotions to explain conspiracy beliefs (Grzesiak‐Feldman, 2013; Kofta et al., 2020; Newheiser et al., 2011; Van Prooijen, 2016; Van Prooijen & Acker, 2015; Whitson & Galinsky, 2008), research suggests that also various positive emotions can increase conspiracy beliefs (Whitson et al., 2015). Emotional intensity (independent of valence) may reconcile these previous findings, and provides a novel perspective on the role of emotions in conspiracy beliefs. Third, Studies 3 to 5 of the current contribution make the novel point that particularly people scoring high on sensation seeking are susceptible to conspiracy theories. These findings extend previous findings that conspiracy beliefs are associated with the narrower construct susceptibility to boredom (Brotherton & Eser, 2015), and contributes to a body of research suggesting that stable individual difference variables predict people’s susceptibility to conspiracy theories (e.g., Cichocka et al., 2016; Swami et al., 2011). Moreover, Study 3 suggests that particularly people high on sensation seeking associate conspiracy theories with entertainment, underscoring the more general point that the link between situational cues and conspiracy theories are often moderated by other contingencies such as individual difference variables.

The present findings also have broader implications for the role of negative affect in human cognition and behaviour. Feelings of anxiety, uncontrollability, and uncertainty often are interpreted as exclusively aversive experiences that people seek to avoid or regulate (e.g., Park, 2010; Van den Bos, 2009). It has been noted previously, however, that emotional intensity can counter the detrimental effects of negative emotions on overall well‐being (e.g., Fujita et al., 1991). As a thought experiment, imagine a study comparing people who have just seen a horror movie (e.g., ‘the Exorcist’) with a neutral control group. We would be quite comfortable to pre‐register the prediction that participants who have watched the horror movie provide higher ratings than the control group on variables such as anxiety, uncontrollability, and uncertainty. This does not mean that watching the movie was an aversive experience, however. On the contrary, people deliberately choose to expose themselves to such frightening experiences because they are entertaining. Many experiences in daily life yield emotions that are not only positive or negative but also intense, which have unique implications for human cognition and behaviour (see also Van Boven et al., 2010).

Of importance, the current propositions do not hold normative implications regarding the value of believing in conspiracy theories: Observing that people find conspiracy theories entertaining does not imply a recommendation to endorse them (as an analogy, some people find using drugs or excessive gambling entertaining, yet we do not recommend those activities either). It is well‐known that conspiracy theories stimulate harmful behaviours, such as vaccine refusals or decreased efforts to reduce one’s carbon footprints (e.g., Jolley & Douglas, 2014a; Van der Linden, 2015). Rather, the present research was designed to shed light on the scientific question what makes conspiracy theories appealing to people, despite their harmful effects.

### Strengths, limitations, and future research

The results across five studies supported a similar conclusion even though we investigated different conspiracy theories in different settings. This is in line with the notion that although conspiracy theories may differ widely in content, belief in such theories is rooted in similar and predictable underlying psychological processes (Douglas et al., 2017; Van Prooijen, 2020; Van Prooijen & Van Vugt, 2018). Furthermore, the studies combined experiments to reveal the effects of conspiracy exposure and entertainment value (Studies 1–3) with cross‐sectional studies to investigate the implications of these findings for a role of sensation‐seeking in conspiracy theories (Studies 4 and 5). Finally, all the studies reported here were well‐powered, and one of the studies was preregistered, suggesting that the current findings are robust and likely to replicate in follow‐up studies.

One limitation of the present studies is that in Studies 1–3, emotional valence and emotional intensity were assessed with general measures of only one item. It is possible that more sophisticated measures of specific, discrete emotions with negative valence (e.g., anger, anxiety) shape conspiracy thinking independent of intensity. Also, the scope of the present findings is yet unclear. For instance, the causal evidence for the link between entertainment appraisals and conspiracy beliefs depends on Study 3, but the emotional content of the entertainment condition may have had additional effects unaccounted for (e.g., in acrimonious social settings, actual corruption may be more likely). Furthermore, it is possible that these findings are moderated by stable individual difference variables. Moreover, entertainment is not the only factor predicting conspiracy belief, and quite often genuine distress (e.g., following social crisis situations such as a pandemic or terrorist attack) stimulates conspiracy thinking (Van Prooijen & Douglas, 2017).

Finally, some conspiracy theories may be very entertaining yet not particularly credible (e.g., flat earth conspiracy theories). One might speculate that for the present effects to occur, entertainment appraisals need to include a serious fascination, rooted in the assumption that a conspiracy theory might be true. If one experiences a less serious form of entertainment (e.g., excessive humour after reading a ridiculous conspiracy theory), the effects observed in the present studies are unlikely to occur. At the same time, we should note that anecdotes exist of clearly fictional stories turning into far‐fetched conspiracy theories that some people genuinely believe. For instance, the ‘alien lizard’ conspiracy theory (assuming that powerful politicians are a breed of alien lizards disguised as humans) shows strong parallels with the plotline of a science fiction series from the 1980s called ‘V’. Likewise, the novel ‘the Da Vinci Code’ has inspired people to believe the conspiracy theory described in the book (Newheiser et al., 2011). Future research may provide a more fine‐grained analysis of the relationships between intense emotional experiences, entertainment appraisals, and conspiracy beliefs.

### Concluding remarks

A growing body of research has underscored the detrimental implications of conspiracy theories for perceivers and society at large. Believing in conspiracy theories is for instance associated with poor health choices, climate change denialism, prejudice, hostility, intergroup conflict, and radicalism (for overviews, see Butter & Knight, 2020; Douglas et al., 2017; Van Prooijen, 2018, 2020; Van Prooijen & Van Vugt, 2018). We deny none of these associations. We do propose, however, that the question of why large groups of citizens believe conspiracy theories despite their negative implications is an important one to answer. The present findings suggest a possible psychological benefit of conspiracy theories to perceivers: Conspiracy theories hold entertainment value, which stimulates belief in them.

## Conflicts of interest

All authors declare no conflict of interest.

## Author contribution

Sabine Rosema (Conceptualization; Formal analysis; Investigation; Methodology) Yang Xu (Conceptualization; Formal analysis; Investigation; Methodology) Jan‐Willem van Prooijen (Conceptualization; Data curation; Formal analysis; Investigation; Methodology; Supervision; Writing – original draft; Writing – review & editing) Joline Ligthart (Conceptualization; Formal analysis; Investigation; Methodology).

## Supporting information


**Supinfo S1** Study 1.

## Data Availability

Anonymised copies of all data and materials are publicly available on the Open Science Framework (https://osf.io/w7ekr/).

## Footnotes

^1^
We also examined the factor structure of this scale in Studies 1–3. In all three studies, a scree plot suggested a one‐factor solution. Full factor analysis results in the OSM (Table S1).

^2^
For Studies 3–5, we provide tables with descriptives and correlations of the measured variables in the OSM (Tables S2–S4).

^3^
We took the liberty of modernizing two items from the original Sensation Seeking Scale (SSS‐V; Zuckerman, 1994). Specifically, we considered the two original items derogatory for sexual minorities, and therefore changed their wording (see the OSM; items 22 and 29).
