# Supplementary material for: The entertainment value of conspiracy theories
Source: Br J Psychol. 2021 Jul 14;113(1):25–48. doi: 10.1111/bjop.12522 (PMC9290699; doi:10.1111/bjop.12522)
Supplement: Supplementary file 1 — Supinfo S1 Study 1. [file BJOP-113-25-s001.docx]

Online Supplementary Materials

For

**The Entertainment Value of Conspiracy Theories**

This document first describes the exact stimulus materials as presented to participants. After that, it provides ancillary analyses (specifically the statistics when excluding participants who failed the manipulation checks in Studies 1 and 2), Table S1 with factor analyses referred to in Footnote 1 of the manuscript, and Tables S2 to S4 providing means, standard deviations, and intercorrelations of the measured variables in Studies 3 to 5.

**Study 1**

In this study, your task will be to closely read an excerpt from an Internet article, and to answer some questions about it.

The article is about **the fire that took place in the Notre Dame Cathedral in Paris, on 15 April 2019**. It does not matter how familiar or unfamiliar you are with this event; we ask you to carefully read the internet article, and form an opinion about it.

Click >> to start with the Internet article.

***Control condition:***

**Here is an Internet article about the fire in the Notre Dame Cathedral, by an unknown writer. Please read it carefully:**

"On 15 April 2019 the tragic fire of the Notre Dame Cathedral in Paris took place. This fire has destroyed the roof, and severely damaged the upper walls of this monumental building. Also, three emergency workers were injured. Directly after the fire, officials started to investigate the causes of the fire. The officials eventually concluded that the fire was not a deliberate act, but was due to technological malfunction during renovation works.

There is little reason to question the official reading of this event. A closer investigation suggests powerful groups are mainly motivated to restore the damage. A fundraising campaign for the restoration works has already raised millions of Euros, and these funds will help to rebuild this Cathedral to its original glory. Furthermore, thanks to the efficient actions of rescue workers, many works of art and religious relics were moved to safety early in the emergency. It is easy to blame an event like this on some unknown or powerful interest group, but the truth is that accidents like this sometimes happen for no other reason than bad luck. Furthermore, the French government has done all it could to determine the causes of this tragedy, in order to prevent such an event from happening again in the future. The Notre Dame fire was a tragic accident, and all the relevant information to understand what happened that day has been shared with the public."

| 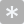 |
| --- |

Q42 Please briefly summarize (in just a few sentences) what the author of this Internet article thinks.

________________________________________________________________

________________________________________________________________

________________________________________________________________

________________________________________________________________

________________________________________________________________

Q49 Does the writer believe that the Notre Dame fire was an accident?

- Yes (1)
- No (2)

***Conspiracy condition:***

Q26 **Here is an Internet article about the fire in the Notre Dame Cathedral, by an unknown writer. Please read it carefully:**

"On 15 April 2019 the tragic fire of the Notre Dame Cathedral in Paris took place. This fire has destroyed the roof, and severely damaged the upper walls of this monumental building. Also, three emergency workers were injured. Directly after the fire, officials started to investigate the causes of the fire. The officials eventually concluded that the fire was not a deliberate act, but was due to technological malfunction during renovation works.

There is, however, ample reason to question the official reading of this event. A closer investigation suggests that various powerful groups benefited in unforeseen ways from the fire. For instance, a fundraising campaign for the restoration works has already raised millions of Euros, and while some of these funds may help to rebuild this Cathedral to its original glory, it is unclear where most of the money is going. Furthermore, it is quite suspicious that many works of art and religious relics were moved to safety early in the emergency; as if powerful groups knew that the fire was about to begin. Around the world there are unknown and powerful interest groups that benefit from incidents like this. Furthermore, the French government has a clear incentive to let the world believe that this was just an accident. It is highly plausible that the Notre Dame was set on fire deliberately, and that the truth about this event is hidden from the public."

| 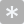 |
| --- |

Q45 Please briefly summarize (in just a few sentences) what the author of this Internet article thinks.

________________________________________________________________

________________________________________________________________

________________________________________________________________

________________________________________________________________

________________________________________________________________

Q50 Does the writer believe that the Notre Dame fire was an accident? (Yes / No)

***Entertainment appraisals measure:***

Q51 **To what extent was the Internet article you just read......**

|  | 1 = Not at all (1) | 2 (2) | 3 (3) | 4 (4) | 5 = Very much (5) |
| --- | --- | --- | --- | --- | --- |
| Interesting (1) |  |  |  |  |  |
| Entertaining (2) |  |  |  |  |  |
| Important (3) |  |  |  |  |  |
| Engaging (4) |  |  |  |  |  |
| Boring (5) |  |  |  |  |  |
| Mysterious (6) |  |  |  |  |  |
| Adventurous (7) |  |  |  |  |  |
| Dull (8) |  |  |  |  |  |
| Captivating (9) |  |  |  |  |  |
| Exciting (10) |  |  |  |  |  |
| Attention-grabbing (11) |  |  |  |  |  |
| Frightening (12) |  |  |  |  |  |

***Emotion measures:***

Q34 Now, please indicate **how positive or negative your emotions were** while reading the article on this slider.

Q35 How positive or negative were the emotions that you felt while reading the article? (ranging from 0 = very negative, to 100 = very positive)

|  | Very Negative | Very Positive |
| --- | --- | --- |

|  | 0 | 10 | 20 | 30 | 40 | 50 | 60 | 70 | 80 | 90 | 100 |
| --- | --- | --- | --- | --- | --- | --- | --- | --- | --- | --- | --- |

| Emotions () | 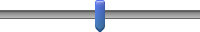 |
| --- | --- |

Q31 Now, please indicate **how intense your emotions were** (regardless of whether they were positive or negative) while reading the article on this slider.

Q33 How intense were the emotions that you felt while reading the article? (ranging from 0 = not at all intense, to 100 = extremely intense)

|  | Not at all intense | Extremely intense |
| --- | --- | --- |

|  | 0 | 10 | 20 | 30 | 40 | 50 | 60 | 70 | 80 | 90 | 100 |
| --- | --- | --- | --- | --- | --- | --- | --- | --- | --- | --- | --- |

| Emotions () | 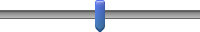 |
| --- | --- |

***Conspiracy belief measure (items 1 to 3):***

Q43 **Please answer the following questions about the text you just read:**

|  | 1 = Not at all (1) | 2 (2) | 3 (3) | 4 (4) | 5 = Very much (5) |
| --- | --- | --- | --- | --- | --- |
| Do you believe that the Notre Dame was set on fire deliberately by a powerful group? (1) |  |  |  |  |  |
| Do you believe that a conspiracy was behind the Notre Dame fire? (2) |  |  |  |  |  |
| Is there reason to be suspicious about the causes of the Notre Dame fire? (3) |  |  |  |  |  |
| Do you agree with the article? (4) |  |  |  |  |  |

(Last item “Do you agree with the article” not reported in the article as it is not part of the conspiracy belief scale and its analysis yielded no additional information).

***Demographics***

Q13 **Before finishing the study, please answer some demographic questions:**

Q14 How would you describe yourself politically?

( 1 = very left wing, 7 = very right-wing)

Q17 Please indicate your gender

- Male (1)
- Female (2)

Q18 What is your age in years?

________________________________________________________________

Q21 What is your education level?

- No formal education (1)
- Primary level education (2)
- Secondary level education (3)
- College education (Bachelor's degree) (4)
- College education (Graduate degree) (5)

**Study 2**

Q25
In this study, your task will be to closely read an excerpt from an Internet article, and to answer some questions about it. 
 
The article is about **the death of the wealthy financier and sex offender Jeffrey Epstein, on 10 August 2019**. It does not matter how familiar or unfamiliar you are with this event; we ask you to carefully read the internet article, and form an opinion about it.
 
Click >> to start with the Internet article.

***Control condition:***

Q25 **Here is an Internet article about the death of Jeffrey Epstein, by an unknown writer. Please read it carefully:**

"On 10 August 2019, the wealthy American financier and convicted sex offender Jeffrey Epstein was found dead in his jail cell. He was found with a bed sheet around his neck, and apparently, he died of suffocation. Epstein was awaiting trial for sex trafficking charges, and for bringing multiple underage girls into his mansion for sexual encounters.  According to official readings, Epstein committed suicide by hanging himself in his jail cell.

There is little reason to question the official reading of this event. Many powerful people wanted him to stand trial and be held accountable for his crimes. Some people believe that he was murdered, but this does not make sense. It is impossible for an assassin to enter and leave a high-security detention center unseen. Everything suggests that Epstein wanted to die, and seized the opportunity. His guards checked upon him every 30 minutes, but when they fell asleep after a long shift, Epstein did not hesitate. The detention center is full of security cameras, which show that nobody entered the wing of Epstein’s cell when it happened. Autopsy results are consistent with the view that Epstein hung himself. Jeffrey Epstein committed suicide in his jail cell, and all the relevant information to understand what happened that night has been shared with the public."

| 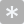 |
| --- |

Q42 Please briefly summarize (in just a few sentences) what the author of this Internet article thinks.

________________________________________________________________

________________________________________________________________

________________________________________________________________

________________________________________________________________

________________________________________________________________

Q49 Does the writer believe that Jeffrey Epstein committed suicide?

- Yes (1)
- No (2)

***Conspiracy condition:***

Q26 **Here is an Internet article about the death of Jeffrey Epstein, by an unknown writer. Please read it carefully:**

"On 10 August 2019, the wealthy American financier and convicted sex offender Jeffrey Epstein was found dead in his jail cell. He was found with a bed sheet around his neck, and apparently, he died of suffocation. Epstein was awaiting trial for sex trafficking charges, and for bringing multiple underage girls into his mansion for sexual encounters. According to official readings, Epstein committed suicide by hanging himself in his jail cell.

There is, however, ample reason to question the official reading of this event. A closer investigation suggests that Epstein trafficked his underage victims to powerful people, who feared a possible testimony. It is surprisingly easy for powerful people to organize an assassination and make it look like suicide, even in a high-security detention center. Everything suggests that Epstein was murdered. His guards were supposed to check upon him every 30 minutes, yet “fell asleep” and left him alone for hours that night. It just so happened that two security cameras overseeing his prison cell conveniently malfunctioned. Autopsy revealed that a bone in his neck has been broken, which more commonly is a result of strangulation rather than hanging. It is highly plausible that Jeffrey Epstein was murdered by powerful and unknown people, and that the truth about this event is hidden from the public."

| 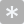 |
| --- |

Q45 Please briefly summarize (in just a few sentences) what the author of this Internet article thinks.

________________________________________________________________

________________________________________________________________

________________________________________________________________

________________________________________________________________

________________________________________________________________

Q50 Does the writer believe that Jeffrey Epstein committed suicide?

- Yes (1)
- No (2)

**Entertainment measure and emotion measures were identical as in Study 1.**

***Conspiracy belief measure (items 1-3):***

Q43 **Please answer the following questions about the text you just read:**

|  | 1 = Not at all (1) | 2 (2) | 3 (3) | 4 (4) | 5 = Very much (5) |
| --- | --- | --- | --- | --- | --- |
| Do you believe that Jeffrey Epstein was murdered by powerful people? (1) |  |  |  |  |  |
| Do you believe that a conspiracy assassinated Jeffrey Epstein? (2) |  |  |  |  |  |
| Is there reason to be suspicious about the death of Jeffrey Epstein? (3) |  |  |  |  |  |
| Do you agree with the article? (4) |  |  |  |  |  |

**As in Study 1 (and for the same reason, item 4 was not included in the manuscript (note that it was also preregistered as exploratory)**

**Demographics were the same as Study 1.**

**Study 3**

Q25 On the next page you will read a description of a country in the world called Contoria. Please read this description carefully.

IMPORTANT: **While reading it, please try to vividly imagine you being a citizen of Contoria**. Imagine that you were born and raised in Contoria, and read the text from the perspective of a citizen of that country.   
 
Click >> to start with the Internet article.

***Entertaining condition:***

**Here is an article about an election in a country called Contoria. Please read it carefully, and vividly imagine you being a citizen of that country:**

Imagine that you live in the country of Contoria. The country has a good social welfare system and the state economy develops rapidly. Contoria also has its fair share of problems, however: There is an uneven distribution of healthcare and educational resources across regions, and particularly in the cities, outdoor air pollution is a problem.

In this country, there is a presidential election between two candidates. The two candidates disagree completely about many issues that are important for the future of Contoria, and they are extremely close to one another in the polls. In debates both candidates passionately argued for their ideas but can’t seem to agree on anything, and regularly they appear to be genuinely angry at each other.

Contorian society is deeply divided between these two candidates, and emotions between citizens are rising high. Quite regularly protests take place, and everyone can feel the tension as two opposing camps are emerging in society. After months of bitter campaigning and conflict, the result of the election is still too close to call and a winner is far from decided. Everyone holds their breath up until the very last minute, as it is extremely unpredictable and exciting who will win.

Q26 Please briefly summarize the events in Contoria (1-3 sentences):

________________________________________________________________

________________________________________________________________

________________________________________________________________

________________________________________________________________

________________________________________________________________

Q42 Did the two candidates become genuinely angry at each other regularly in debates?

- Yes (4)
- No (5)

Q49 Is Contorian society deeply divided because of this election?

- Yes (1)
- No (2)

***Boring condition:***

**Here is an article about an election in a country called Contoria. Please read it carefully, and vividly imagine you being a citizen of that country:**

Imagine that you live in the country of Contoria. The country has a good social welfare system and the state economy develops rapidly. Contoria also has its fair share of problems, however: There is an uneven distribution of healthcare and educational resources across regions, and particularly in the cities, outdoor air pollution is a problem.

In this country, there is a presidential election between two candidates. The two candidates have different positions about effective governance, and seem to have a comparable basis of electoral support. In debates both candidates exchanged their ideas of what legislation and law-making institutions are in need of refinement, and made it apparent that they have different viewpoints on these issues.

Contorian society faces a difficult choice between these two candidates, and citizens often discuss their different points of view of what governmental reform is necessary. Quite regularly groups of citizens publicly articulate their opinions, and everyone tries to resolve the question which candidate proposes the best policies to address societal challenges. After months of campaigning, based on opinion polls it is difficult to ascertain what the outcome of the electoral process will be. Soon it will become known which candidate’s policy program will be implemented.

Q28 Please briefly summarize the events in Contoria (1-3 sentences):

________________________________________________________________

________________________________________________________________

________________________________________________________________

________________________________________________________________

________________________________________________________________

Q45 Did the candidates make it apparent that they have different viewpoints on many issues in debates?

- Yes (4)
- No (5)

Q50 Does Contorian society face a difficult choice between these two candidates?

- Yes (1)
- No (2)

**Measures of entertainment appraisals, emotional valence, and emotional intensity was the same as Studies 1 and 2**

***Conspiracy belief measure:***

Q43 **While answering the following questions, please continue imagining that you are a citizen of Contoria.**

Please indicate how likely or unlikely you consider it that the following issues occur: (1 = very unlikely, 5 = very likely)

|  | 1 = very unlikely (1) | 2 (2) | 3 (3) | 4 (4) | 5 = very likely (5) |
| --- | --- | --- | --- | --- | --- |
| There will be cheating in the results counting process. (1) |  |  |  |  |  |
| Election officers are bribed to favor one of the candidates. (2) |  |  |  |  |  |
| Secret organizations in Contoria strongly influence the election outcome. (3) |  |  |  |  |  |
| Opinion polls have been secretly manipulated. (4) |  |  |  |  |  |
| The winner has already been decided in secret before the election. (5) |  |  |  |  |  |
| There are “shadowy forces” behind the elections. (6) |  |  |  |  |  |
| A conspiracy will determine the election outcome. (7) |  |  |  |  |  |

***Sensation seeking measure (Hoyle, 2002)***

On the following screen, we will ask you a few questions about your preferences in life. After that, we will ask for some demographics.

| Page Break |  |
| --- | --- |

Q35 Please indicate to what extent do you agree with the following statements: (1 = strongly disagree, 5 = strongly agree)

|  | 1= strongly disagree (1) | 2 (6) | 3 (7) | 4 (4) | 5 = strongly agree (10) |
| --- | --- | --- | --- | --- | --- |
| I would like to explore strange places (1) |  |  |  |  |  |
| I get restless when I spend too much time at home. (2) |  |  |  |  |  |
| I like to do frightening things. (3) |  |  |  |  |  |
| I like wild parties. (4) |  |  |  |  |  |
| I would like to take off on a trip with no pre-planned routes or timetables. (5) |  |  |  |  |  |
| I prefer friends who are excitingly unpredictable. (6) |  |  |  |  |  |
| I would like to try bungee jumping. (7) |  |  |  |  |  |
| I would love to have new and exciting experiences, even if they are illegal. (8) |  |  |  |  |  |

**Study 4**

Q93 In the first section of the questionnaire we ask you to fill in two general questions about the organization you are currently working in, and a couple of background questions.

Q79 How long have you been working in your current organization? (in months)

________________________________________________________________

Skip To: End of Survey If Condition: How long have you been work... Is Less Than 3. Skip To: End of Survey.

Q80 How many people work for your current organization?

________________________________________________________________

Skip To: End of Survey If Condition: How many people work for yo... Is Less Than 15. Skip To: End of Survey.

Q76 What is your gender?

- Male (1)
- Female (2)

Q100 How old are you?

________________________________________________________________

Q98 Would you consider yourself religious?

- Not at all (1)
- A little (2)
- Somewhat (3)
- Quite a bit (4)
- Very much (5)

Q99 What is your religion?

________________________________________________________________

Q101 What is your highest level of completed education?

- No formal education (1)
- Primary level education (2)
- Secondary level education (3)
- College education (Bachelor's degree) (4)
- College education (Graduate degree) (5)

Q102 What is your ethnicity?

- Caucasian (1)
- African American (2)
- Native American (3)
- Asian American (4)
- Native Hawaiian or Pacific Islander (5)
- Hispanic / Latino (6)
- Other (7)

Q103 How would you describe yourself politically, on a scale from 1 (very left-wing) to 11 (very right-wing)

Q104 Do you consider yourself to be a Democrat or a Republican?

(1 = Clearly Democrat, 5 = Clearly Republican; 6 = Neither Democrat nor Republican)

***Sensation seeking measure:***

Q42 You finished the section with the general questions and the questions about the organization you are currently working at. 


 The following questions will be about what kind of experiences you prefer. 


Each of the items contains two choices: A and B. Please choose the option that most describes your likes or the way you feel. In some cases you may find items in which both choices describe your likes or feelings. Please choose the one which better describes your likes or feelings.


In some cases you may find items in which you do not like either choice. In these cases mark the choice you dislike least. Please try to answer each item.

Q1 Choice 1

- A. I like "wild" uninhibited parties
- B. I prefer quiet parties with good conversation

Q2 Choice 2

- A. There are some movies I enjoy seeing a second or even a third time
- B. I can't stand watching a movie I've seen before

Q3 Choice 3

- A. I often wish I could be a mountain climber
- B. I can't understand people who risk their necks climbing mountains

Q4 Choice 4

- A. I dislike all body odors
- B. I like some for the earthy body smells

Q5 Choice 5

- A. I get bored seeing the same old faces
- B. I like the comfortable familiarity of everyday friends

Q6 Choice 6

- A. I like to explore a strange city or section of town by myself, even if it means getting lost
- B. I prefer a guide when I am in a place I don't know well

Q7 Choice 7

- A. I dislike people who do or say things just to shock or upset others
- B. When you can predict almost everything a person will do and say he or she must be a bore

Q8 Choice 8

- A. I usually don't enjoy a movie or play where I can predict what will happen in advance
- B. I don't mind watching a movie or a play where I can predict what will happen in advance

Q9 Choice 9

- A. I have tried marijuana or would like to
- B. I would never smoke marijuana

Q10 Choice 10

- A. I would not like to try any drug which might produce strange and dangerous effects on me
- B. I would like to try some of the new drugs that produce hallucinations

Q11 Choice 11

- A. A sensible person avoids activities that are dangerous
- B. I sometimes like to do things that are a little frightening

Q12 Choice 12

- A. I dislike "swingers" (people who are uninhibited and free about sex)
- B. I enjoy the company of real "swingers"

Q13 Choice 13

- A. I find that stimulants make me uncomfortable
- B. I often like to get high (drinking liquor or smoking marijuana)

Q14 Choice 14

- A. I like to try new foods that I have never tasted before
- B. I order the dishes with which I am familiar, so as to avoid disappointment and unpleasantness

Q15 Choice 15

- A. I enjoy looking at home movies or travel slides
- B. Looking at someone's home movies or travel slides bores me tremendously

Q16 Choice 16

- A. I would like to take up the sport water skiing
- B. I would not like to take up water skiing

Q17 Choice 17

- I would like to try surfboarding
- I would not like to try surfboarding

Q18 Choice 18

- I would like to take off on a trip with no preplanned or definite routes, or timetable
- B. When I go on a trip I like to plan my route and timetable fairly carefully

Q19 Choice 19

- A. I prefer the "down to earth" kinds of people as friends
- B. I would like to make friends in some of the "far out" groups like artists or "punks"

Q20 Choice 20

- A. I would not like to learn to fly an airplane
- B. I would like to learn to fly an airplane

Q21 Choice 21

- A. I prefer the surface of the water to the depths
- B. I would like to go scuba diving

Q22 Choice 22

- A. I would like to meet some persons who are really different than I am
- B. I stay away from anyone who I suspect being really different from me

Q23 Choice 23

- A. I would like to try parachute jumping
- B. I would never want to try jumping out of a plane with or without a parachute

Q24 Choice 24

- A. I prefer friends who are excitingly unpredictable
- B. I prefer friends who are reliable and predictable

Q25 Choice 25

- A. I am not interested in experience for its own sake
- B. I like to have new and exciting experiences and sensations even if they are a little frightening, unconventional, or illegal

Q26 Choice 26

- A. The essence of good art is in its clarity, symmetry of form and harmony of coulors
- B. I often find beauty in the "clashing" colors and irregular forms of modern paintings

Q27 Choice 27

- A. I enjoy spending time in the familiar surroundings of home
- B. I get very restless if I have to stay around home for any length of time

Q28 Choice 28

- A. I like to dive off the high board
- B. I don't like the feeling I get standing on the high board (or I don't go near it at all)

Q29 Choice 29

- A. I like to date people who are physically exciting
- B. I like to date people who share my values

Q30 Choice 30

- A. Heavy drinking usually ruins a party because some people get loud and boisterous
- B. Keeping the drinks full is the key to a good party

Q31 Choice 31

- A. The worst social sin is to be rude
- B. The worst social sin is to be a bore

Q32 Choice 32

- A. A person should have considerable sexual experience before marriage
- B. Its better if two married persons begin their sexual experience with each other

Q33 Choice 33

- A. Even if I had the money I would not care to associate with flight rich persons like those in the "jet set"
- B. I could conceive of myself seeking pleasures around the world with the "jet set"

Q34 Choice 34

- A. I like people who are sharp and witty even if they do sometimes insult others
- B. I dislike people who have their fun at the expense of hurting the feelings of others

Q35 Choice 35

- A. There is altogether too much portrayal of sex in movies
- B. I enjoy watching many of the "sexy" scenes in movies

Q36 Choice 36

- A. I feel best after taking a couple of drinks
- B. Something is wrong with people who need liquor to feel good

Q37 Choice 37

- A. People should dress according to some standard of taste, neatness, and style
- B. People should dress in individual ways even if the effects are sometimes strange

Q38 Choice 38

- A. Sailing long distances in small sailing crafts is foolhardy
- B. I would like to sail a long distance in a small but seaworthy sailing craft

Q39 Choice 39

- A. I have no patience with dull or boring persons
- B. I find something interesting in almost every person I talk to

Q40 Choice 40

- A. Skiing down a high mountain slope is a good way to end up on crutches
- B. I think I would enjoy the sensations of skiing very fast down a high mountain slope

***Organizational conspiracy beliefs (answered on a scale 1 = strongly disagree, 5 = strongly agree):***

Q81 You finished the questions about what kind of experiences you prefer. 

In the next couple of questions we would like to know more about how you think/feel about your management.

Q43 Our management has a hidden agenda

Q52 Our management had hidden goals which benefit only them

Q53 I suspect that our managers frequently lie to employees about important issues

Q54 Our managers would never consciously hide important information from us employees

Q55 Our supervisors would never conspire against subordinates

Q56 Our managers gossip about subordinates behind their backs

Q57 Our supervisors work together to achieve a hidden agenda that they deliberately keep secret

Q58 Our supervisors pass on confidential data regarding us employees to one another

Q59 Our supervisors try to achieve hidden, malevolent goals

***Conspiracy mentality (1 = certainly not, 11 = certainly)***

Q82 You finished the questions about how you think/feel about your management.


The following questions will be about how you think about events in the world more generally.

- I think that many very important things happen to the world, which the public is never informed about
- I think that politicians usually do not tell us the true motives for their decisions
- I think that government agencies closely monitor all citizens
- I think that events which superficially seem to lack a connection are often the result of secret activities
- I think that there are secret organizations that greatly influence political decisions

***Following the conspiracy mentality measure the questionnaire included measures of work satisfaction, organizational commitment, and a single-item question asking for absenteism. We regard these variables as less central to the current research and therefore do not address them in the paper.***

**Study 5**

**Demographics and Sensation Seeking Scale were the same as in Study 4.**

***Conspiracy beliefs scale (1 = Definitely not true, 5 = Definitely true):***

Q81 You finished the questions about what kind of experiences you prefer. In the next couple of questions we would like to know more about how you think/feel about some topics.

There is often debate about whether or not the public is told the whole truth about various important issues. These questions are designed to assess your beliefs about some of these subjects. Please indicate the degree to which you believe each statement is likely to be true.

CB1 The US government deliberately conceals a lot of information from the public

CB2 Ebola is a man-made virus

CB3 The US government had advance knowledge of the 9/11 attacks

CB4 The US government covered up crucial information in the aftermath of J. F. Kennedy’s assassination

CB5 The science behind global warming has been invented or distorted out of self-interest

CB6 Various wars in the Middle East were launched by oil companies

CB7 The moon landing was a hoax

CB8 The HIV/aids virus has been genetically engineered to wipe out certain sectors of the population

CB9 Evidence of unidentified flying objects and extraterrestrial visitors is being suppressed by the government

**Measure of conspiracy mentality was the same as in Study 4.**

**Ancillary analyses**

***Study 1***

In Study 1, 16 participants failed the manipulation check (10 in the control condition, 6 in the conspiracy condition). Here are the main test statistics when these 16 participants are excluded from the data:

Main effect condition on entertainment appraisals: *F*(1, 284) = 65.01, *p* < .001; ω^2^ = .18, CI_95%_[.11; .27]

Main effect condition on emotional valence: *F*(1, 283) = 55.39, *p* < .001; ω^2^ = .16, CI_95%_[.09; .24]

Emotional valence as covariate in the analysis of emotional intensity: *F*(1, 281) = 3.963, *p* = .047; ω^2^ = .01, CI_95%_[.00; .05]

Main effect condition while controlling for emotional intensity: *F*(1, 281) = 11.69, *p* < .001; ω^2^ = .04, CI_95%_[.01; .09]

Main effect condition on conspiracy belief: *F*(1, 282) = 95.152, *p* < .001; ω^2^ = .25, CI_95%_[.16; .34]

Indirect effect through entertainment appraisals: *B* = 0.17, *SE* = .08; CI_95%_[0.02; 0.33]

Indirect effect through emotional intensity: *B* = 0.07, *SE* = .03; CI_95%_[0.01; 0.15]

Indirect effect through emotional valence: *B* = -0.02, *SE* = .06; CI_95%_[-0.14; 0.10]

***Study 2***

In Study 1, 35 participants failed the manipulation check (16 in the control condition, 19 in the conspiracy condition). Here are the main test statistics when these 35 participants are excluded from the data:

Main effect condition on entertainment appraisals: *F*(1, 265) = 75.49, *p* < .001; ω^2^ = .22, CI_95%_[.13; .31]

Main effect condition on emotional valence: *F*(1, 265) = 3.928, *p* = .049; ω^2^ = .01, CI_95%_[.00; .05]

Emotional valence as covariate in the analysis of emotional intensity: *F*(1, 264) = 1.163, *p* = .282; ω^2^ = .00, CI_95%_[.00; .02]

Main effect condition while controlling for emotional intensity: *F*(1, 264) = 15.147, *p* <.001; ω^2^ = .05, CI_95%_[.01; .11]

Main effect condition on conspiracy belief: *F*(1, 265) = 20.502, *p* < .001; ω^2^ = .07, CI_95%_[.02; .14]

Indirect effect through entertainment appraisals: *B* = 0.24, *SE* = .09; CI_95%_[0.04; 0.43]

Indirect effect through emotional intensity: *B* = 0.02, *SE* = .05; CI_95%_[-0.08; 0.11]

Indirect effect through emotional valence: *B* = -0.05, *SE* = .03; CI_95%_[-0.12; -0.01]

**Table S1**

Factor loadings of the entertainment appraisals measure (1-factor solution, Principal Axis Factoring) across studies

*Study 1 Study 2 Study 3*

Interesting .795 .822 .871

Entertaining .705 .656 .772

Important .543 .714 .620

Engaging .824 .841 .855

Boring -.675 -.583 -.713

Mysterious .567 .627 .382

Adventurous .638 .595 .675

Dull -.631 -.557 -.641

Captivating .836 .852 .855

Exciting .776 .774 .837

Attention-grabbing .825 .806 .885

Frightening .487 .542 .357

*Eigenvalue* 5.905 5.991 6.334

**Table S2**

Means, Standard Deviations, and Intercorrelations of the Measured Variables in Study 3.

*M SD* 1 2 3 4 5

1. Entertainment appraisals 2.77 0.85 -

2. Emotional intensity 38.32 26.51 .63^***^ -

3. Emotional valence 46.20 20.75 .28^***^ .10^*^ -

4. Belief in conspiracy theories 2.25 1.08 .15^***^ .15^**^ -.08 -

5. Sensation seeking 2.60 0.84 .03 -.03 .06 .12^**^ -

*Note.* Entertainment appraisals, belief in conspiracy theories, and sensation seeking were measured on 5-point scales, emotional valence and emotional intensity on 100-point scales. Higher means represent higher scores on the variable in question. ^*^ *p* < .05; ^**^ *p* < .01; ^***^ *p* < .001.

**Table S3**

Means, Standard Deviations, and Intercorrelations of the Measured Variables in Study 4.

*M SD* 1 2 3 4 5 6 7

1. SSS 17.22 5.82 -

2. Boredom susceptibility 3.54 2.22 .59^***^ -

3. Disinhibition 3.93 2.13 .74^***^ .31^***^ -

4. Experience seeking 5.01 1.87 .62^***^ .08 .38^***^ -

5. TAS 4.74 2.52 .70^***^ .15^**^ .31^***^ .30^***^ -

6. OCB 3.04 0.77 .34^***^ .36^***^ .22^***^ .09 .22^***^ -

7. Conspiracy mentality 7.02 2.39 -.11 -.24^***^ -.07 -.04 .04 .09 -

*Note.* SSS = Sensation Seeking Scale; TAS = Thrill and Adventure Seeking; OCB = Organizational Conspiracy Beliefs. Sensation Seeking was measured on a scale from 0 to 40; Boredom Susceptibility, Disinhibition, Experience Seeking, and Thrill and Adventure Seeking were measured on a scale from 0 to 10; Organizational Conspiracy Beliefs on a scale from 1 to 5; and Conspiracy mentality on a scale from 1 to 11. ^**^ *p* < .01; ^***^ *p* < .001.

**Table S4**

Means, Standard Deviations, and Intercorrelations of the Measured Variables in Study 5.

*M SD* 1 2 3 4 5 6 7

1. SSS 16.69 6.87 -

2. Boredom susceptibility 3.47 1.82 .61^***^ -

3. Disinhibition 4.03 2.44 .74^***^ .38^***^ -

4. Experience seeking 5.12 2.24 .73^***^ .28^***^ .44^***^ -

5. TAS 4.06 3.03 .76^***^ .28^***^ .32^***^ .40^***^ -

6. Conspiracy beliefs 2.69 0.85 .13^**^ .11^*^ .11^*^ -.02 .15^**^ -

7. Conspiracy mentality 7.44 2.10 .08 .02 .11^*^ .02 .07 .71^***^ -

*Note.* SSS = Sensation Seeking Scale; TAS = Thrill and Adventure Seeking. Sensation Seeking was measured on a scale from 0 to 40; Boredom Susceptibility, Disinhibition, Experience Seeking, and Thrill and Adventure Seeking were measured on a scale from 0 to 10; Conspiracy Beliefs on a scale from 1 to 5; and Conspiracy mentality on a scale from 1 to 11. ^*^ *p* < .05; ^**^ *p* < .01; ^***^ *p* < .001.
